# Improving emergency management of status epilepticus: a randomized high-fidelity simulator-based trial

**DOI:** 10.1007/s00415-026-14058-8

**Published:** 2026-08-19

**Authors:** Paulina S. C. Kliem, Kai Tisljar, Sira M. Baumann, Pascale Grzonka, Gian Marco De Marchis, Stefano Bassetti, Roland Bingisser, Sabina Hunziker, Stephan Marsch, Raoul Sutter

**Affiliations:** 1 Clinic for Intensive Care Medicine, Intensive Care Units, University Hospital Basel, Petersgraben 4, 4031 Basel, Switzerland; 2 Department of Anesthesiology and Intensive Care Medicine, Canton Hospital of Basel-Landschaft, Basel, Switzerland; 3 Department of Neurology, University Teaching and Research Hospital, HOCH-Cantonal Hospital St, Gallen, Switzerland; 4 Medical Faculty, University of Basel, Basel, Switzerland; 5 Division of Internal Medicine, University Hospital Basel, Basel, Switzerland; 6 Department of Emergency Medicine, University Hospital Basel, Basel, Switzerland; 7 Department of Psychosomatic Medicine, University Hospital Basel, Basel, Switzerland

**Keywords:** Status epilepticus, Guidelines, Neurocritical care, Simulator study, Randomized trial

## Abstract

**Purpose:**

Evaluating how different status epilepticus (SE) guideline formats affect physicians’ management of SE in high-fidelity simulation. The primary objective was adherence to guideline-recommended diagnostic and treatment interventions; the secondary was guideline memorability.

**Materials and methods:**

A randomized controlled trial was conducted, randomizing physicians from various specialties at the University Hospital Basel, a Swiss academic care center into three groups: (1) without guidelines (controls), (2) with access to NCS guidelines, (3) provided with a condensed single-page guideline incorporating symbols to facilitate information assimilation and evaluation. Each group performed an identical simulated SE scenario. Primary endpoints included vital sign checks, therapeutic interventions, and timing. Secondary endpoints assessed questionnaire-based aftermath self-evaluation and guideline memorability.

**Results:**

Among 124 participating physicians, 35% were controls, 34% provided with NCS guideline, and 31% with the condensed single-page guideline. Participants using the condensed guideline showed higher adherence to key interventions, with significantly improved rates of side positioning for airway protection (63% with condensed guideline vs. 25% in controls and 26% in the full guideline group; p<0.001) and second-line antiseizure drug administration (97% vs. 70% and 86%; p=0.003). No significant differences were observed in vital sign checks or other interventions. The condensed guideline was rated as more memorable compared to the original guidelines (*p*=0.001).

**Conclusions:**

Condensed SE guidelines improved adherence to selected emergency interventions compared with both full NCS guidelines and no guideline support. Guideline usability may represent a modifiable target for improving time-critical neurological emergencies.

**Trial Registration:**

Clinicaltrials.gov ID NCT03883516 2019-03-12.

**Supplementary Information:**

The online version contains supplementary material available at https://doi.org/10.1007/s00415-026-14058-8.

## Introduction

Status epilepticus (SE) constitutes a critical neurological emergency characterized by substantial morbidity, mortality, and associated socioeconomic burden [1–6]. Its management necessitates prompt intervention encompassing emergency measures, such as airway management, oxygenation, stabilization of metabolic and hemodynamic parameters, prevention of physical injury, etiological investigation and treatment, and the timely administration of antiseizure drugs (ASDs). Addressing these challenges, current international treatment guidelines for SE, as established by the Neurocritical Care Society (NCS), delineate specific sequential therapeutic interventions [7]. Despite these efforts, a systematic review has revealed that delayed or incorrect administration of ASDs is reported in up to 60% across multiple studies [8]. Following this systematic review, subsequent studies repeatedly demonstrated deviations from established SE treatment guidelines. Our 10-year retrospective cohort study of out-of-hospital adult SE patients revealed infrequent benzodiazepine administration especially in elderly patients with altered consciousness at onset [9]. Concordantly, the Italian prospective STEPPER study reported only 63% adherence to SE guidelines, highlighting significant benzodiazepine underutilization (71%), particularly in in-hospital onset SE [10]. This deviation correlated with increased treatment failure, mortality, and functional impairment [10]. A review of pediatric SE management further revealed substantial benzodiazepine administration delays (mean 25 min), non-adherent to recommendations and again linked to poorer outcomes [11]. A German multicenter registry retrospective study reported initial benzodiazepine treatment in only 72% of SE patients, with adequate dosing in only 22% [12]. These findings are in line with the results from our previous high-fidelity simulator-based studies on a highly standardized clinical scenario of SE [13, 14]. In our first study, only 54% of participating physicians checked airways and a mere 16% protected them [13]. Supplementary oxygen was administered by 76%. In our second simulator-based study, physicians systematically checked all vital signs as recommended by the guidelines in only 5% [14].

Despite this evidence of significant deviations from SE management guidelines, standardized studies evaluating specific interventions to optimize guideline adherence in emergency settings are scarce. High-fidelity simulation provides such a standardized clinical environment for detailed investigation of clinical practice and the impact of guideline implementation, as demonstrated extensively in cardiopulmonary resuscitation research [15–17].

This study aimed to investigate the impact of providing different formats of SE treatment guidelines (i.e., the complete standard treatment guidelines [7] versus a condensed, single-page guideline) on the quality of emergency management by physicians within a randomized controlled trial utilizing high-fidelity simulation.

## Materials and methods

### Setting, study design, and randomization

This investigator-initiated randomized high-fidelity simulator-based trial was performed between December 2020 and July 2024 at the simulation center of the intensive care unit (ICU) at the University Hospital Basel, a Swiss academic tertiary care center. As in our prior studies, workshops to train clinicians in the management of a simulated emergency scenario were offered to medical doctors working as resident physicians in different medical fields (e.g., intensive care medicine, emergency medicine, internal medicine, and neurology). The training was offered during regular working hours without additional compensation to all residents in participating specialties. Participants lacked prior training regarding SE diagnosis and management. Pre-simulation, all participants completed a questionnaire detailing demographics, medical knowledge, specialization, prior simulation experience, clinical experience, and pre-simulation work hours.

**Fig. 1:**
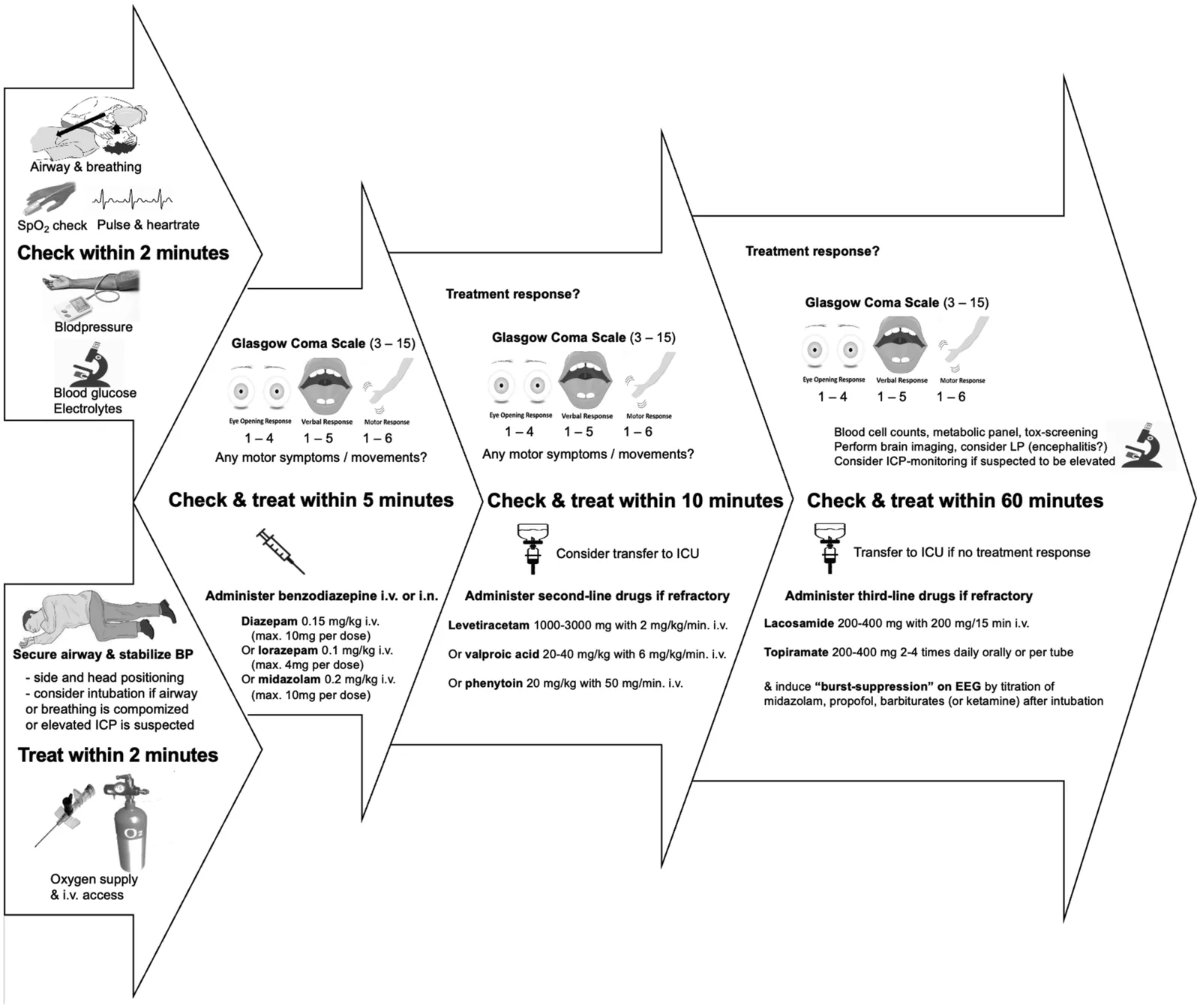
Condensed, single-page guideline summarizing the pivotal recommendations of the management of status epilepticus according to the Neurocritical Care Society [7] incorporating symbolic representations designed to facilitate rapid information assimilation and evaluation

Prior to the training in the high-fidelity simulator, all physicians were randomized by random numbering by the principal investigator (R.S.) to the following three groups: 1. physicians not provided with any SE treatment guidelines (i.e., controls); 2. physicians provided with the original SE treatment guidelines as provided by the NCS; and 3. physicians provided with a condensed, single-page guideline summarizing the pivotal recommendations of the NCS [7] incorporating symbolic representations designed to facilitate rapid information assimilation and evaluation as presented in Fig. 1. Participants assigned to groups 2 and 3 were allotted a 5-min period immediately before entering the simulated scenario to carefully review the provided standard treatment guidelines (with important and relevant recommendations marked yellow) or the condensed, single-page guideline, respectively. Prior to the study initiation, the study team had selectively marked key recommendations of the official NCS treatment guidelines to facilitate and accelerate the information retrieval. The 5-min allocation was established as an adequate timeframe for reviewing all highlighted recommendations based on a preliminary pilot trial. In this pilot trial, five non-neurologist physicians with no prior exposure to the NCS guidelines successfully reviewed the complete set of highlighted recommendations within the specified 5-min limit as confirmed by the physicians. Participants were not explicitly asked if they had managed to read all relevant information in either guideline that was provided. Upon completion of the review period, the guidelines were collected from the physicians.

We adhered to the STrengthening the Reporting of OBservational studies in Epidemiology (STROBE) guidelines to enhance the quality of the study [18].

### High-fidelity simulator setup

High-fidelity simulation center equipment, as detailed in our previous studies [13, 14], utilized a programmable mannequin (SimMan®, Laerdal Medical AS, Stavanger, Norway) capable of simulating diverse physiological and pathological states (vocalization, pulses, breathing sounds and movements, eye/pupil changes, motor activity including tonic–clonic movements, secretions including the production of foamy sputum, enuresis). Bedside monitors displayed dynamic vital signs contingent on applied interventions and only if monitoring was established by the participating physicians. Comprehensive emergency medications (i.e., vasopressors, antimicrobials, steroids, thiamine, fluids, glucose, first/second-line ASDs, and anesthetics), intubation/suction equipment, flashlights, and dressing materials were available. The mannequin was equipped with an intravenous access line. An instructed and pre-trained nurse assisted with diagnostics and monitoring upon the participants’ requests and acted strictly command driven and not spontaneously.

### Simulated clinical SE scenario

The simulated scenario of an adult patient in minimally convulsive SE due to alcohol withdrawal was the same as used for a prior study [13]. All participants were informed that the patient was admitted with SE, that they would be the physician on duty in an emergency department and that a nurse would be available to assist them upon request. They further received a standardized introduction to the simulator room technique prior to simulation. All participants performed the identical scenario individually. Prior to the training, physicians were randomized to the three groups as defined above.

At the start of the scenario, the participating physician was called to ED for a patient with minimal convulsions for several minutes. An unremarkable computed tomogram and an unremarkable cerebrospinal fluid analysis (performed due to unexplained loss of consciousness) were displayed. The paramedic report (printed records) described a neighbor hearing noises and the patient being found on floor. Laboratory results revealed normoglycemia, lactic acidosis (in an otherwise unremarkable blood gas analysis), negative toxicologic screening, a macrocytosis in blood analysis, and elevated liver enzymes. The scenario ended 1 min following the administration of at least one first- and one second-line ASD, or spontaneously after 20 min if no ASDs were administered (with a patient regaining consciousness within 1 min). Post-scenario, physicians were asked to complete a self-assessment using an emotional reflexivity questionnaire as presented in the *Supplemental *Table 1 and to rate guideline memorability on a Likert scale from 0 to 10 with 0 being not memorable and 10 being very memorable.

**Table 1:** Baseline characteristics of participating physicians categorized by randomization groups (*n*=124)

Baseline characteristics of participants	Participants without guidelines (controls; *n*=44)		Participants with NCS guidelines (*n*=42)		Participants with one-page summary guidelines (*n*=38)		*p* value
*Demographics*							
Age (years; median, IQR)	31	29–33	32	30–34	32	30–35	0.562
Female (*n*, %)	27	61.4	27	64.3	24	63.2	0.972
*Professional baselines*							
Physicians’ affiliations							
Intensive care/emergency medicine (*n*, %)	19	43.2	18	42.9	15	39.5	0.892
Internal medicine (*n*, %)	11	25.0	14	33.3	12	31.6	
Neurology (*n*, %)	14	31.8	10	23.8	11	29.0	
Years of clinical experience within the current affiliation (years; median, IQR)	4.0	2.5–5.0	4.6	3.0–6.0	4.0	2.5–5.0	0.418
Previous simulator training (*n*, %)	17	38.6	22	52.4	17	44.7	0.426
Working hours prior to participation (hours; median, IQR)	9.0	0.0–10.0	9.0	0.0–10.0	9.5	0.0–10.5	0.602
Subjective stress level prior to training (range 1–10; median, IQR)	5	4–7	5	3–6	4	3–6	0.503

*SE* status epilepticus, *NCS* neurocritical care society, *IQR* interquartile range

### Outcomes and measurements

Primary endpoints were performed checks of vital signs (including airways, breathing, oxygenation, pulse, heart rate, and blood pressure) and their timing, laboratory data review, and therapeutic interventions (including non-invasive airway protection with head and/or side positioning to avoid aspiration and airway obstruction or loss, or invasive airway protection by endotracheal intubation, administration of oxygen and first- and second-line ASDs) and differences across the three groups.

Secondary endpoints were post-simulation self-evaluation and subjective scenario-related rating in the debriefing and differences across the three groups.

### Data assessment

Participants’ performances and simulated vital signs were frame-in-frame video/audio recorded. Two independent observers coded primary endpoints second-by-second from recordings. Interrater agreement for categorical variables was assessed using Cohen’s kappa (κ); continuous variables (time to action) were compared. In cases with an interrater disagreement, the videotapes were jointly reviewed until consensus was reached.

### A priori sample size calculations

Based on the assumption of a 50% absolute increase in adherence to the treatment guideline attributable to our intervention—an effect size informed by a simulation-based study [19] investigating the impact of simulation-based education on the quality of care during cardiac arrest team responses at an academic teaching hospital—power analysis indicated that 14 simulations per group were required to achieve a two-sided significance level (α) of 0.05 and a power (1-β) of 0.9. To account for potential attrition and ensure sufficient statistical power, we aimed to recruit at least 20 physicians per group, each participating in one simulation, resulting in a minimum total of 60 simulations across the 3 study arms.

### Statistics

Participants were categorized into three groups according to their randomized exposure to the NCS SE treatment guidelines with group 1 representing participants not provided with any guidelines (i.e., controls), group 2 representing participants provided with the official NCS treatment guidelines, and group 3 representing participants provided with a condensed, single-page guideline summarizing the pivotal recommendations of the Neurocritical Care Society [7]. Discrete variables are expressed as counts (percentage) and continuous variables are expressed as medians and interquartile ranges (IQR). Univariable comparisons of categorical variables among the three groups defined above were performed using the Fisher’s exact test. Continuous variables were compared among the three groups using the Kruskal–Wallis equality-of-populations rank test. Two-sided *p* values were considered significant at different *p* values depending on the Bonferroni corrections for multiple comparisons [20]. Statistical analysis was performed with STATA®16.1 (Stata Corp., College Station, TX, USA).

### Standard protocol approvals, registration, and consents

This study was registered at ClinicalTrials.gov prior to initiation (ID NCT03883516). The study was approved by the institutional ethics committee and written informed consent was obtained from all participants (Ethikkommission Nordwest- und Zentralschweiz [EKNZ] No. 2019–00168).

### Use of artificial intelligence-assisted technologies

The large language model ChatGPT versions 4omni to 5.6 (OpenAI, San Francisco, USA) were used to paraphrase and summarize some of the manuscript’s content. After using this application, the authors further edited the content as needed and verified its accuracy.

### Data availability statement

The corresponding author has full access to all the data in the study. He takes full responsibility for the integrity of the data, the accuracy of the data analysis and interpretation, and the conduct of the research. The authors have the right to publish any and all data, separate and apart from the guidance of any sponsor.

The study protocol and statistical analysis plan were published in advance at clinicaltrials.gov. A pilot study [13] (outlined in the protocol as “first study”) to test the reliable recognition of SE by the participants has already been published.

## Results

### Cohort description

In total, 128 simulation trainings were recorded, 42 of the participants had access to NCS guidelines and 41 were provided with a condensed single-page guideline incorporating symbols to facilitate information assimilation and evaluation. Four recordings were excluded due to technical problems (*Supplemental Fig. 1*)*.* Univariable comparisons of baseline characteristics of all 124 participants categorized by randomization groups are presented in Table 1*.* Comparisons revealed no significant differences regarding age, sex, affiliations, years of clinical experience, previous simulator training with other scenarios, working hours, and subjective stress level. Males were slightly underrepresented, consistent with the current percentage of males in medical education in Switzerland (BAG Statistiken Ärztinnen/Ärzte, 2019). Stated working hours agreed with the Swiss labor law.

Interrater agreement regarding categorical variables was κ=0.92. SE was recognized by all participants.

### Diagnostic and treatment performance (primary endpoints)

Univariable comparisons of diagnostic performance characteristics of participating physicians categorized by randomization groups are presented in Table 2. Comparisons revealed no significant differences among the three groups. Although, at first glance, breathing and heart rate checks were more frequently performed within the recommended 2 min by group 3 (i.e., physicians who read the condensed, single-page guideline), these differences were no longer significant after Bonferroni correction for multiple comparisons (with a significance threshold set at a *p*≤0.003).

**Table 2:** Diagnostic performance characteristics of participating physicians categorized by randomization groups (*n*=124)

Performance characteristics of participants	Participants without guidelines (controls; n=44)		Participants with NCS guidelines (n=42)		Participants with one-page summary guidelines (n=38)		*p* value*
SE diagnosis questioned (*n*, %)	0	0.0	2	4.8	0	0.0	0.205
Patient history checked (*n*, %)	36	81.8	38	90.5	33	86.8	0.520
Time to first history check (minutes; median, IQR)	2.3	1.0–3.8	2.1	0.7–3.7	3.0	1.6–4.6	0.235
*Vital signs check*							
All vital signs checked (*n*, %)	37	84.1	31	73.8	27	71.1	0.308
All vitals checked within 2 min (*n*, %)	17	38.6	10	23.8	15	39.5	0.229
Airways checked (*n*, %)	32	72.7	24	57.1	26	68.4	0.325
Airwiays checked within 2 min (*n*, %)	20	45.5	17	40.5	19	50.0	0.672
Breathing checked (*n*, %)	33	74.0	29	69.1	29	76.3	0.764
Breathing checked within 2 min (*n*, %)	21	47.7	11	26.2	19	50.0	0.050
Oxygenation checked (*n*, %)	43	97.7	41	97.6	38	100.0	1.000
Oxygenation checked within 2 min (*n*, %)	38	86.4	37	88.1	36	94.7	0.469
Heart rate checked (*n*, %)	43	97.7	39	92.9	36	94.7	0.595
Heart rate checked within 2 min (n, %)	31	70.5	20	47.6	27	71.1	0.050
Blood pressure checked (*n*, %)	41	93.2	39	92.9	37	97.4	0.701
Blood pressure checked within 2 min (n, %)	28	63.6	24	57.1	30	79.0	0.109
*Laboratory investigations checked*							
Laboratory investigations checked (n, %)	38	86.4	41	97.6	35	92.1	0.159
Laboratory investigations checked within 2 min (n, %)	8	18.2	14	33.3	10	26.3	0.288
Calling for EEG (*n*, %)	3	6.8	4	9.5	1	2.6	0.482

*SE* status epilepticus; *IQR* inter quartile range; *EEG* electroencephalography

^*^ Level of significance was set at p≤0.003 after Bonferroni correction for multiple comparisons

Univariable comparisons of treatment performance characteristics across the randomization groups are detailed in Table 3. Although the majority of treatment steps exhibited higher frequency in group 3 compared to groups 1 and 2, and group 1 (i.e., controls) demonstrated the lowest proportion of participants performing most treatment steps relative to groups 2 and 3, only side positioning for airway protection and the administration of second-line ASDs showed statistically significant higher frequency of performance in group 3 following Bonferroni correction for multiple comparisons (with a significance threshold set at a *p*≤0.003).

**Table 3:** Treatment performance characteristics of participating physicians categorized by randomization groups (*n*=124)

Performance characteristics of participants	Participants without guidelines (controls; *n*=44)		Participants with NCS guidelines (*n*=42)		Participants with one-page summary guidelines (*n*=38)		*p* value*
*Treatment measures*							
Side positioning for airway protection (*n*, %)	11	25.0	11	26.2	24	63.2	**<0.001**
Time to side positioning within 2 min (*n*, %)	8	18.2	7	16.7	5	13.2	0.828
Oxygen supply (*n*, %)	39	88.6	38	90.5	36	94.7	0.673
Time oxygen supply within 2 min (*n*, %)	14	31.8	17	40.5	19	50.0	0.253
Call for intubation (*n*, %)	6	13.6	4	9.5	6	15.8	0.750
Time to call for intubation (minutes; median, IQR)	12.0	10.7–13.0	8.7	7.5–11.2	5.8	4.2–9.3	0.033
ASDs administered overall (*n*, %)	44	100.0	42	100.0	38	100.0	NA
Number of ASDs administered overall (median, IQR)	2	2–2	2	2–2	2	2–2	0.587
Benzodiazepines administered as first ASD (*n*, %)	43	97.7	41	97.6	38	100.0	1.000
Time to first benzodiazepine (minutes; median, IQR)	2.4	1.5–3.8	2.1	1.4–4.1	3.3	2.2–4.1	0.146
Different benzodiazepines mixed (*n*, %)	9	20.5	6	14.3	1	2.6	0.039
Second-line ASD administered (*n*, %)	31	70.5	36	85.7	37	97.4	**0.003**
Time to second-line ASD (minutes; median, IQR)	5.5	3.7–9.8	6.3	4.7–11.3	7.5	6.6–10.8	0.126
Anesthetics continuously administered (*n*, %)	3	6.8	0	0.0	0	0.0	0.107
Time to continuous anesthesia (minutes; median, IQR)	11.1	7.7–13.0	NA		NA		NA
Calling for additional staff (*n*, %)	30	68.2	32	76.2	23	60.5	0.331
*Treatment response check and observation*							
Treatment response checked (*n*, %)	41	93.2	36	85.7	36	94.7	0.323
Time to treatment response check (minutes, median, IQR)	4.5	2.8–7.4	5.2	3.4–7.7	5.8	3.6–7.9	0.455

*SE* status epilepticus, *IQR* inter quartile range, *ASD* antiseizure drug; *EEG* electroencephalography

^*^Bold font indicates significance after Bonferroni correction for multiple comparisons (with a level of significance set at *p*≤0.003)

### Aftermath self-evaluation (secondary endpoints)

Table 4 presents the aftermath characteristics regarding self-evaluation and scenario-reporting as provided by the participating physicians after the simulator training. At first glance, the subjective evaluation of the quality of the physicians’ own performances was rated to be higher in group 3 as compared to groups 1 and 2; however, the association was not significant after correction for multiple comparisons (with a significance threshold set at a *p*≤0.006). However, not surprisingly, analysis of the participants’ evaluations of the provided guidelines indicated that physicians receiving the condensed, single-page guideline reported a significantly higher degree of memorability compared to those receiving the full original guidelines.

**Table 4:** Aftermath characteristics regarding self-evaluation and scenario-reporting (*n*=124)

Aftermath self-evaluation (ranging from 1 to 10 with 1 representing the lowest and 10 the highest level)	Participants without guidelines (controls; *n*=44)		Participants with NCS guidelines (*n*=42)		Participants with one-page summary guidelines (*n*=38)		*p* value*
Subjective stress level during training (median, IQR)	7	5–8	7	6–7	7	5–7	0.758
Subjective motivation during training (median, IQR)	9	7–10	9	8–10	9	8–10	0.787
Subjective challenge felt during training (median, IQR)	8	7–8	8	7–8	7	7–8	0.523
Subjective certainty during training (median, IQR)	6	4–8	7	5–8	6	5–7	0.370
Subjective quality of own performance during training (median, IQR)	5	5–6	6	5–7	7	5–7	0.024
Subjective quality of team performance (median, IQR)	8	7–9	9	8–10	9	8–9	0.075
Subjective certainty with diagnosis (median, IQR)	8	7–10	8	7–10	9	7–10	0.664
Aftermath evaluation of presented guidelines (ranging from 1 to 10 with 1 representing the lowest and 10 the highest level)							
Memorability of guidelines (median, IQR)	NA	NA	8	7–10	9	8–10	**0.001**

*NCS* neurocritical Care Society, *IQR* interquartile range

Data assessed in the aftermath of the training by asking the participants to self-evaluate using the emotional reflexivity questionnaire (Supplemental Table 1)

^*^Bold font indicates significance after Bonferroni correction for multiple comparisons (with a level of significance set at *p*≤0.006)

## Discussion

This randomized controlled trial utilizing a highly standardized simulated clinical scenario of SE in a high-fidelity simulator aimed to investigate the impact of providing different formats of SE treatment guidelines on the quality of emergency management by resident physicians.

Our findings concerning treatment steps showed, after correcting for multiple comparisons, that side positioning for airway protection and the administration of second-line ASDs was significantly more frequent in the condensed guideline group compared to the participants with original full NCS guidelines and controls. Regarding the participant feedback, the physicians exposed to the condensed guideline self-reported a statistically significant increase in guideline memorability compared to the controls and physicians confronted with the original guidelines. While self-reporting may not reliably reflect objective behavior [21], assessment of participants’ guideline adherence during the simulated scenario also supported single-page guideline use as it was associated with greater adherence to key treatment steps.

As revealed in our previous simulator-based study, critical interventions, such as airway management (16% performance rate) and the administration of second-line ASDs following the failure of first-line treatment with benzodiazepine (57% performance rate), are frequently suboptimal, potentially increasing patients’ risks of adverse outcomes [13].

Suboptimal management of patients exhibiting altered consciousness, particularly concerning airway assessment and protection, raises significant concerns regarding heightened patient vulnerability to aspiration events and subsequent pneumonia, as well as influences mortality; the former complication being of clinical relevance in the context of SE, as respiratory tract infections have been implicated in the evolution of SE to treatment-refractory SE, a condition associated with increased mortality rates [5, 22–24].

The observation that nearly one-third of physicians without access to treatment guidelines in our trial failed to escalate toward second-line ASD in benzodiazepine-refractory SE is concerning and unfortunately aligns with results from our prior simulation-based studies on SE [13] and multiple clinical investigations correlating the absence or delay in second-line ASD escalation with poorer neurological outcomes [8, 25, 26].

In 2024, the American College of Emergency Physicians published their own guideline, solely focusing on the question which second-line therapy should be administered without mentioning any other diagnostic or therapeutic recommendations. They conclude that levetiracetam, valproate, and fosphenytoin have a similar risk profile and will result in the cessation of seizure in half the patients, stressing that early treatment results in a reduction of morbidity and mortality [27]. Current reviews focusing on how to improve the “status quo” in SE management also emphasize the rapid escalation to second-line treatments [28, 29], discussing optimization in standardized protocols, SE order sets as well as provider education to potentially shorten the time to both first- and second-line therapy [30].

Our findings suggest that guideline complexity itself may represent a modifiable system factor influencing emergency care performance. Evidence regarding the effect of abbreviated guidelines in other emergency scenarios is scarce. However, previous work suggests that simplifying the presentation of recommendations may improve their implementation in time-critical settings. For example, in 2023, a quality improvement study with a pre–post-intervention comparison revealed a higher adherence to antibiotic prescription guidelines in the emergency department following the introduction of a simplified guideline supporting antibiotic stewardship [31]. In addition, the principles of condensed, rapidly accessible guidance are already used in several other areas of emergency medicine, such as ambulance services and the guidelines by the European Society of Cardiology [32], incorporating symbolic or abbreviated presentations to facilitate rapid information retrieval and clinical decision-making.

The finding that a condensed, single-page guideline incorporating symbolic representations designed to facilitate rapid information assimilation and evaluation improved the frequency of specific critical interventions, namely airway protection via side positioning and the administration of second-line ASDs, has therefore potentially important implications for clinical practice.

Persistent challenges in timely and appropriate SE management as documented in prior studies highlight the urgent need for strategies that improve adherence to established guidelines.

Our findings show that presenting complex recommendations in a simplified, readily accessible and thereby more memorable format may facilitate their effective implementation—especially for critical and time-sensitive interventions crucial in the early management of SE.

Various tools such as the Reporting Tool for Practice Guidelines (RIGHT) [33] offer a framework to improve clarity and usability of clinical practice guidelines. For instance, its structured approach supports redesign efforts like the National Guideline for Field Triage after Emergency Medical Services feedback, showing how transparent reporting can enhance adoption and effectiveness in real-world clinical practice [34].

Our findings support incorporating principles from cognitive psychology, human factors engineering, and information design into guideline development for time-critical neurological emergencies. Although Large Language Models (LLMs) may increasingly be used as clinical decision-support tools, our previous study showed that, for example, when asking ChatGPT regarding emergency SE management, it recommended several key interventions in SE management inconsistently and depending on the type of prompting [35]. Together with our current findings, this suggests that human-centered guideline design remains important.

Our findings have several clinical implications. First, concise, user-friendly guidelines with visual aids and clear recommendations could improve adherence to guidelines and impact treatment in acute neurological emergencies, especially for less-experienced clinicians and in time-sensitive situations. Second, guideline format influences memorability—future designs should apply cognitive psychology and information design principles aided by guideline evaluation tools. Third, while simulation studies are useful and training in simulation centers represents a valuable additional key component to increase the quality of emergency management in clinical practice, real-world research is needed to confirm their impact on patient outcomes. Implementing condensed guidelines alongside education, simulation training, and feedback could enhance care for critical neurological conditions.

### Limitations

This study has some limitations. The single-center design may limit generalizability. However, as most physicians are trained in multiple different Swiss and international medical care centers during their curriculum, this shortcoming is likely to be very limited. Furthermore, despite its high standardization, the simulator setting may have only limited transferability and generalizability, and our results need confirmation in real life. However, high-fidelity simulation is commonly used as a central training tool for advanced life support [36] and our scenario proved to be realistic since the identical simulation was tested in two prior “pilot” studies and by the fact that all participants recognized SE. An additional limitation involves the potential for self-reporting bias during the subsequent subjective evaluation, wherein participants self-reported the perceived memorability of the guidelines.

## Conclusion

This simulation-based randomized controlled trial suggests that a condensed, single-page SE treatment guideline improves adherence to key interventions, such as airway protection and second-line ASD administration, compared to traditional guidelines. The enhanced memorability of the simplified format may further aid clinicians in high-pressure scenarios. However, since not all aspects of SE management improved, a multifaceted approach—combining concise guidelines, simulation training, and real-world implementation—is needed to optimize care. Further studies in clinical settings are necessary to validate these findings and assess their impact on patient outcomes.

## Supplementary Information

Below is the link to the electronic supplementary material.

Supplementary file1 (PDF 49 kb)

Supplementary file2 (PDF 153 kb)

## Data Availability

The datasets used and/or analyzed during the current study are available from the corresponding author on reasonable request. Restrictions apply to the availability of the data to guarantee the anonymity of the volunteering physicians.
