# Supplementary material for: Improving emergency management of status epilepticus: a randomized high-fidelity simulator-based trial
Source: J Neurol. 2026 Aug 19;273(9):533. doi: 10.1007/s00415-026-14058-8 (PMC13490203; doi:10.1007/s00415-026-14058-8)
Supplement: Supplementary file 1 [file 415_2026_14058_MOESM1_ESM.pdf]

Supplemental Table 1: Emotional reflexivity questionnaire for subjective self-evaluation after completing the simulated clinical scenario

**How was your stress level during the simulation?**									
☐ 1	☐ 2	☐ 3	☐ 4	☐ 5	☐ 6	☐ 7	☐ 8	☐ 9	☐ 10
Not stressed					***Severely stressed***				
**How was your motivation during the simulation?**									
☐ 1	☐ 2	☐ 3	☐ 4	☐ 5	☐ 6	☐ 7	☐ 8	☐ 9	☐ 10
Not motivated					***Strongly motivated***				
**How challenging was the simulation?**									
☐ 1	☐ 2	☐ 3	☐ 4	☐ 5	☐ 6	☐ 7	☐ 8	☐ 9	☐ 10
Not at all					***Very much***				
**How certain were you?**									
☐ 1	☐ 2	☐ 3	☐ 4	☐ 5	☐ 6	☐ 7	☐ 8	☐ 9	☐ 10
Uncertain					***Certain***				
**How good was your clinical performance?**									
☐ 1	☐ 2	☐ 3	☐ 4	☐ 5	☐ 6	☐ 7	☐ 8	☐ 9	☐ 10
Not good					***Excellent***				
**How good was the performance of the team?**									
☐ 1	☐ 2	☐ 3	☐ 4	☐ 5	☐ 6	☐ 7	☐ 8	☐ 9	☐ 10
Not good					***Excellent***				
**How certain were you regarding the diagnosis?**									
☐ 1	☐ 2	☐ 3	☐ 4	☐ 5	☐ 6	☐ 7	☐ 8	☐ 9	☐ 10
Uncertain					***Certain***				
