# Supplementary material for: Improving emergency management of status epilepticus: a randomized high-fidelity simulator-based trial
Source: J Neurol. 2026 Aug 19;273(9):533. doi: 10.1007/s00415-026-14058-8 (PMC13490203; doi:10.1007/s00415-026-14058-8)
Supplement: Supplementary file 2 [file 415_2026_14058_MOESM2_ESM.pdf]

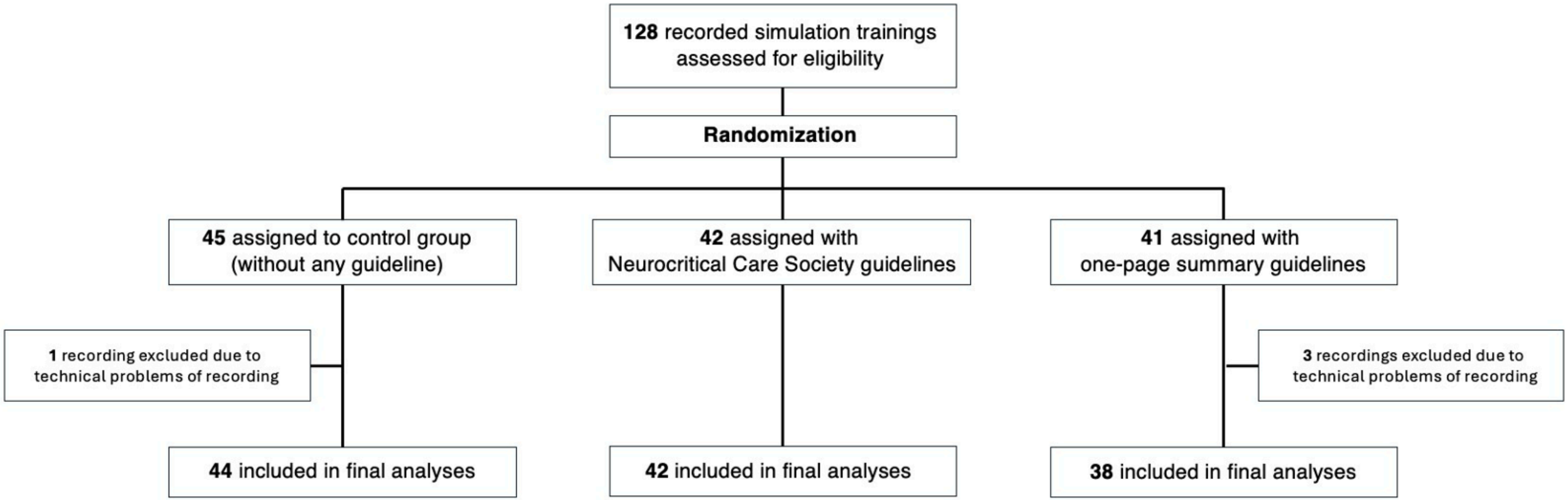

Suppl. Fig. 1 Flow Chart
